# Exploring how weight stigma relates to psychological distress, physical activity, and eating behaviors over time: a longitudinal study among young adults in Hong Kong

**DOI:** 10.1186/s40337-026-01525-w

**Published:** 2026-01-19

**Authors:** Xavier C. C. Fung, Andrew M. H. Siu, Jiajia Ye, Jian-Han Chen, Jung-Sheng Chen, Nadia Bevan, Mark D. Griffiths, Chung-Ying Lin, Benson W. M. Lau

**Affiliations:** 1 Department of Rehabilitation Sciences, Faculty of Health and Social Sciences, The Hong Kong Polytechnic University, Hung Hom, Hong Kong, China; 2 Sau Po Centre on Ageing, The University of Hong Kong, Pokfulam, Hong Kong, China; 3 Department of Health Sciences, College of Health, Medicine, and Life Sciences, Brunel University London, London, UK; 4 Department of Rehabilitation assessments, Rehabilitation Hospital Affiliated to Fujian University of Traditional Chinese Medicine, Fuzhou, China; 5 Fujian Key Laboratory of Rehabilitation Technology, Fuzhou, Fujian China; 6 Bariatric and Metabolism International Surgery Center, E-Da Hospital, I-Shou University, Kaohsiung, Taiwan; 7 Department of General Surgery, E-Da Hospital, I-Shou University, Kaohsiung, Taiwan; 8 Department of Medical Research, E-Da Hospital, I-Shou University, Kaohsiung, Taiwan; 9 School of Social Sciences, Faculty of Arts, Monash University, 20 Chancellors Walk, Clayton, VIC 3800 Australia; 10 Psychology Department, Nottingham Trent University, 50 Shakespeare St, Nottingham, NG1 4FQ UK; 11 Institute of Allied Health Sciences, College of Medicine, National Cheng Kung University, 1 University Road, Tainan, 701401 Taiwan; 12 Biostatistics Consulting Center, National Cheng Kung University Hospital, College of Medicine, National Cheng Kung University, Tainan, Taiwan; 13 Department of Public Health, College of Medicine, National Cheng Kung University, Tainan, Taiwan; 14 School of Nursing, College of Nursing, Kaohsiung Medical University, Kaohsiung, Taiwan

**Keywords:** Weight stigma, Eating behaviors, Physical activity, Psychological distress, Young adults, Longitudinal study

## Abstract

**Background:**

Many researchers have expressed concerns that weight stigma may cause adverse health effects and worsen weight issues in a vicious cycle. However, empirical evidence evaluating this cycle is scarce, especially among Eastern Asians. The present study investigated the temporal associations among perceived weight stigma, weight-related self-stigma, psychological distress, perceived behavioral control, physical activity, eating behaviors, and body mass index (BMI) changes.

**Methods:**

A one-year longitudinal survey was carried out to explore if the weight cycle exists among young adults in Hong Kong. The study comprised 345 participants at Time 1 (T_1_), 253 participants at T_2_, 233 participants at T_3_, and 235 participants at T_4_. Participants completed self-reported psychometric instruments in an online survey. The analysis employed parallel process latent growth curve modeling and a random intercept cross-lagged panel model.

**Results:**

Temporal relationships existed in the connections between perceived stigma and self-stigma, and self-stigma and perceived behavioral control. A negative relationship between self-stigma and future BMI was found, whereas future self-stigma showed no significant association with previous BMI.

**Conclusion:**

The growth trajectories of the studied variables did not correlate with changes in BMI. However, self-stigma showed a negative association with subsequent BMI in a different model. Further research is needed to clarify whether weight stigma is impacted by changes in BMI.

**Supplementary Information:**

The online version contains supplementary material available at 10.1186/s40337-026-01525-w.

## Abstract (plain language summary)

Weight stigma, discrimination due to an individual’s body weight, is a public health concern. It has been found that weight stigma is harmful to an individual’s mental and physical health, and such negative impact may create a harmful cycle. However, there is little clear evidence that the cycle exists, especially among individuals from East Asia. To investigate this issue, a study followed young adults in Hong Kong to examine how weight stigma relates to mental health, eating behaviors, exercise habits, and weight changes. Young adults were surveyed over a one-year period across four timepoints; Time 1 (T_1_; *n* = 345), T_2_ (*n* = 253), T_3_ (*n* = 233), T_4_ (*n* = 235). Results indicated that relationships existed over time in the connections between (i) perceived stigma (awareness of stigma) and self-stigma (stigma towards oneself), and (ii) between self-stigma and perceived behavioral control. In summary, the rate of change of these variables did not correlate with changes in body mass index (BMI). However, self-stigma showed a negative association with BMI at later timepoints. Findings supported a clearer understanding of weight management and highlighted the harmful impact of stigma. The findings also showed significant issues with weight stigma in Hong Kong, emphasizing the need for improved public education and stigma reduction efforts. Further research is needed to determine whether weight stigma can be influenced by changes in weight.

**Supplementary Information:**

The online version contains supplementary material available at 10.1186/s40337-026-01525-w.

## Introduction

Weight stigma denotes the social devaluation and discrimination encountered by individuals as a result of their body weight, frequently manifesting through negative stereotypes, prejudice, or unfair treatment [1]. Weight stigma is pervasive and affects individuals across various settings such as healthcare, education, and workplaces, as well as within personal relationships and through media portrayals [2]. An international study found that more than 50% of participants across Australia, Canada, France, Germany, the United Kingdom, and the United States had experienced weight stigma [3]. A report from the Hong Kong Obesity Society indicated that the general perception of individuals with higher weight in Hong Kong was negative [4]. In a survey of 559 participants, 67% believed that individuals with higher weight were lazy when it comes to exercising, 62% thought they frequently engaged in binge eating, and over 50% felt they lacked willpower [4]. Moreover, studies on weight stigma in Hong Kong (where the present study was conducted) have highlighted its negative impact on quality of life, leading to unhealthy behaviors, diminished health-promoting behaviors, and emotional challenges [5–8].

Weight stigma, reflecting social attitude and stereotypes, is closely associated with other individual stigma concepts such as perceived weight stigma (PWS) and weight-related self-stigma (WRSS). WRSS refers to individuals internalizing adverse stereotypes associated with body weight, whereas PWS refers to an individual’s awareness or belief that they are being judged or experiencing discrimination from others because of their weight. Both forms of stigma are associated with adverse psychological and behavioral outcomes, such as increased psychological distress, uncontrolled eating, emotional eating, and reduced physical activity (PA) [9–11]. Understanding weight stigma is crucial for developing interventions to mitigate its pervasive effects and managing its impact on individual health and well-being.

Weight stigma has a significant impact on perceived behavioral control, especially concerning weight management and health behaviors. Research suggests that exposure to weight stigma may boost individuals’ motivation for weight-loss but concurrently reduce their perceived ability to achieve weight-loss and may result in an increase in unhealthy weight-loss behaviors [12]. In other words, perceived behavioral control plays a significant role in the relationship between stigma and weight management behaviors. For instance, WRSS may negatively influence perceived behavioral control and engagement in PA, leading to a greater difficulty in maintaining a healthy lifestyle [7]. Similarly, WRSS may significantly influence the intention to avoid inappropriate eating behaviors among young adults, as well as reducing their perceived control over avoiding such behaviors [8]. In addition, it has been found that perceived control is associated with psychological distress [13–15]. Therefore, it is also worth examining whether the reduced perceived behavioral control resulting from WRSS can lead to psychological distress.

Weight stigma has also been found to be associated with psychological distress (e.g., poorer mental health outcomes) [9]. A meta-analysis showed moderate pooled associations between PWS and depression, and between WRSS and psychological distress, including depression and anxiety [9]. Another review study indicated that PWS is related to psychological distress, with WRSS acting as a potential mediator, although further evidence is needed to confirm this [16].

The literature has hypothesized that weight stigma may form a vicious cycle in relation to poor weight management behaviors and weight gain [17, 18]. More specifically, stigma-induced stress and psychological distress can lead to maladaptive eating and stigma avoidance behaviors [17, 18]. In turn, maladaptive eating and avoidance behaviors, such as avoiding physical activity [19], can lead to weight gain and/or hinder weight loss efforts [17]. Consequently, increased weight gain can heighten stigma and create a vicious cycle [17, 18]. Moreover, whether BMI is associated with future WRSS remains a critical gap in understanding this vicious cycle. This has not been well investigated in previous research and requires empirical exploration. To obtain a comprehensive understanding of the stigma and its consequences, the stage model of self-stigma on mental illness proposed by Corrigan and Rao [20] is a valuable framework. This model describes how self-stigma forms and its subsequent effects. When applied to the context of weight stigma, the stage model of self-stigma has also been discussed as effective in exploring the relationships between weight stigma and the related consequences [21]. The model proposed how weight stigma could impact weight management behavior in four stages: awareness, agreement, application, and harm. Awareness involves recognizing negative beliefs, stereotypes, or stigmas in society. Next, individuals may agree with these beliefs and thoughts and apply them to understand and explain their own daily experiences. The personal application of negative beliefs can lead to psychological harm, such as diminishing self-esteem or perceived ability. This stage model explains how self-stigma may trigger a “why try effect”, hindering individual efforts in weight management [20]. Moreover, a study conducted by Prunty et al. [22] reported that weight stigma is widespread across individuals of all body sizes. Therefore, the model may be universally applicable because every individual can potentially experience weight stigma.

**Fig. 1:**
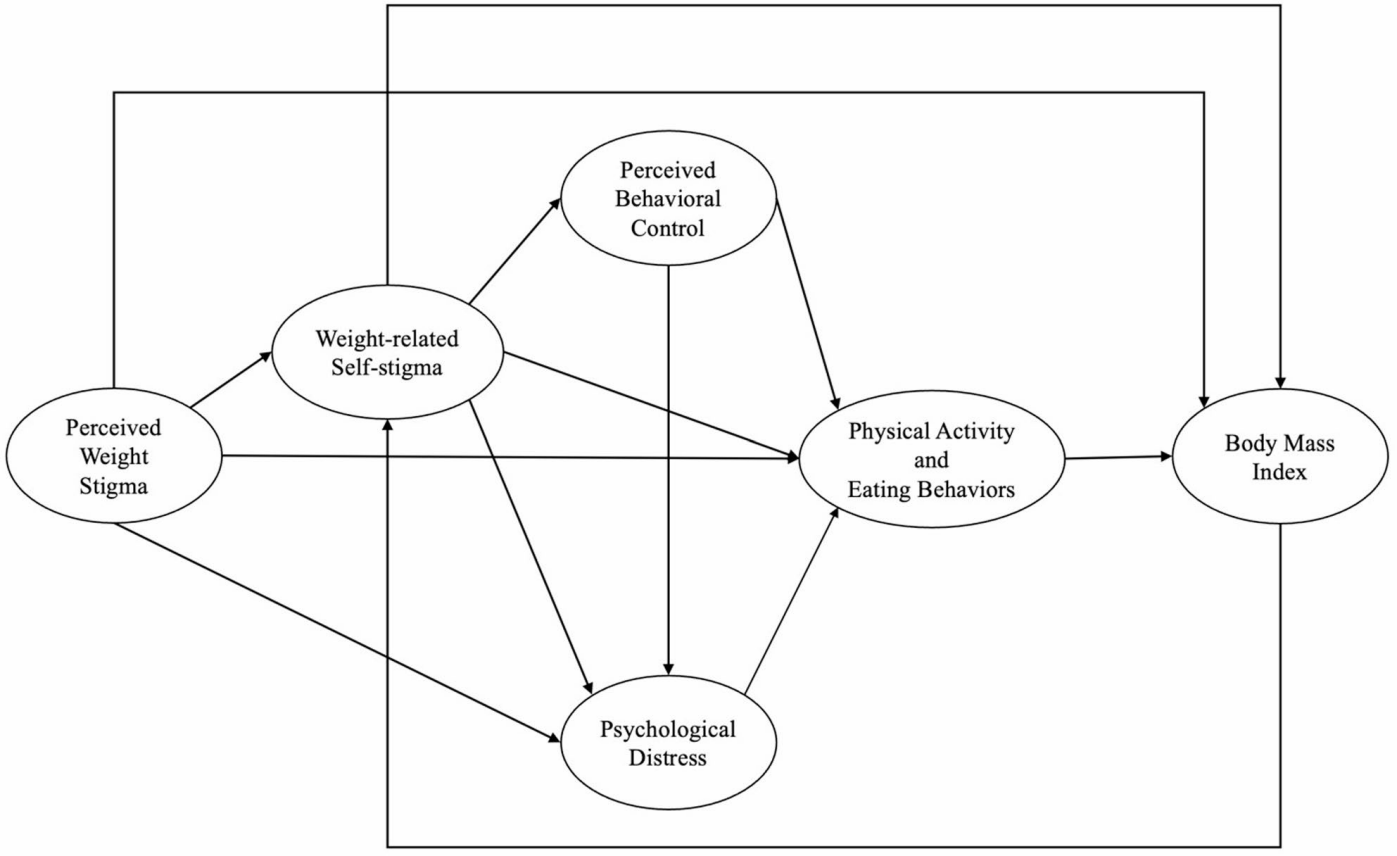
The conceptual model of weight stigma process

The present study adopted this stage model to explore the impact of weight stigma over time. The model specifically applies PWS as a key variable indicating awareness, WRSS as an indicator of agreement and application, and perceived behavioral control as an indicator of harm. Expanding on this model, and supported by previous literature, the present study integrated psychological distress, weight management behaviors (including PA and eating behaviors), and body mass index (BMI) as variables influenced by weight stigma (see Fig. 1). The study also utilized various analyses for the conceptual model to explore the process from stigma development to BMI change, as well as the potential reciprocal relationship between WRSS and BMI. More specifically, the present study employed a parallel-process latent growth curve model (PP-LGCM) to examine the impact of stigma, focusing on the growth trajectories over time. Additionally, a random intercept cross-lagged panel model (RI-CLPM) was applied to assess how fluctuations in WRSS and BMI within individuals can influence each other’s changes at multiple timepoints.

In the present study, a one-year longitudinal survey was conducted to address two research objectives. Firstly, to investigate how PWS, WRSS, psychological distress, perceived behavioral control, PA, and eating behaviors interact over time. Secondly, to explore the reciprocal relationship between WRSS and changes in BMI throughout a longitudinal study. It was hypothesized that: (i) PWS would have a positive temporal association with WRSS (H_1_); (ii) WRSS would have negative temporal associations with perceived behavioral control (H_2_); (iii) PWS and WRSS would have negative temporal associations with psychological distress, (H_3_); (iv) perceived behavioral control would be negatively associated with psychological distress, emotional eating, uncontrolled eating, and positively related to PA and cognitive restraint over time (H_4_); (v) psychological distress would be negatively associated with PA and eating behaviors over time (H_5_); (vi) WRSS would be associated with PA negatively and eating behaviors positively, with both influencing the BMI over time (H_6_), and (vii) WRSS and BMI would be associated with one another across time (H_7_).

## Methods

As part of a wider study [23], the present study explored perceived weight stigma, self-stigmatization related to weight, perceived behavioral control, psychological distress, eating behaviors, and PA among young adults. The longitudinal study was not pre-registered and was conducted between March 2021 to June 2022 and obtained ethical approval from the Ethics Review Board of the Hong Kong Polytechnic University (HSEARS20201120002). Participants were requested to complete a set of psychometric instruments within an online survey over four timepoints across 12 months.

### Participants

The participants were young adults from a Hong Kong university recruited using convenience sampling. Participants were between 18 and 30 years of age, proficient in Traditional Chinese, and provided informed consent to participate. Exclusion criteria included self-reported diagnoses of neurological disorders, functional impairments, or psychotic conditions that could interfere with completing online surveys, such as difficulties using electronic devices, or conditions affecting outcomes such as physical activity and eating. To encourage continued participation and prevent dropout in the longitudinal study, participants received HK$50 for completing at least two waves, along with an additional HK$200 for those who completed all four waves.

Two participants were excluded from analysis because they fell outside the specified age range, and three others were removed from the dataset for providing invalid PA data, e.g., reporting more than 16 hours of activity per day, indicating no activity yet reporting a duration or reporting engagement in activity but stated zero minutes [24]. A total of 345 valid participants were enrolled in the first wave of data collection. During the second wave, 253 of these individuals participated (follow-up rate of 73.3%). In the third wave, 233 participants from the first wave responded to the survey (67.5% follow-up rate), while 234 participants responded to the fourth wave (follow-up rate of 67.8%).

### Measures

The survey was designed to collect demographic information and data on key variables comprising weight stigma, psychological distress, perceived behavioral control, physical activity, and eating behaviors.

#### Demographic data

Participants were requested to provide demographic data, including age, sex, height, and weight. Moreover, considering the reported impact of COVID-19 on PA and eating behaviors, including decreased levels of PA [25], increased total food intake [26], and dietary changes [27], participants were asked if they considered COVID-19 had affected their PA and eating behaviors (i.e., more, the same, or less) over the past month. This question was included as a covariate in the analysis (please refer to the ‘Data analysis’ section).

#### Perceived weight stigma (PWS)

The Perceived Weight Stigma Scale (PWSS) developed by Lin et al. [28] was used to assess perceived experience of stigmatized situations due to weight. It comprises ten dichotomous items, where a score of 0 indicates no perceived stigma, and a score of 1 indicates the presence of such experiences. An example item is “*People treat you less politely than others because of your weight status.*” Total scores are obtained by adding all item responses, where higher scores reflect greater PWS. The Chinese version of the PWSS has shown satisfactory internal consistency, evidenced by a Cronbach’s alpha of 0.84 [28]. Moreover, the scale’s psychometric properties underwent further validation through confirmatory factor analysis, showing a unidimensional factor structure with acceptable fit indices based on a sample of participants from Hong Kong [28]. In the present study, Cronbach’s α for the PWS ranged from 0.82 to 0.88 across T_1_ to T_4_.

#### Weight-related self-stigma (WRSS)

The Weight Bias Internalization Scale (WBIS), developed by Durso and Latner [29], was used to assess WRSS. This instrument was later modified by Pearl and Puhl [30] to be applicable to individuals of diverse weight statuses, such as both non-overweight and overweight. This 11-item instrument utilized a 5-point rating scale, with higher scores indicating increased internalization of weight-related stigma. An example item is “*I am less attractive than most other people because of my weight.*” The Chinese adaptation of the WBIS, which adjusted item descriptions to suit various weight statuses, has shown satisfactory psychometric properties among both populations with overweight and non-overweight [31]. The internal consistency of the WBIS, as assessed by Cronbach’s α, ranged from 0.90 to 0.91 across T_1_ to T_4_ in the present study. Additionally, based on T_1_ scores, participants were divided into low and high WRSS groups using the recommended cutoff, defined as the scale’s midpoint [32].

#### Physical activity (PA)

The International Physical Activity Questionnaire (IPAQ) developed by Craig et al. [33] was used to assess PA. The present study used the Chinese short form with seven items [34]. The IPAQ assesses the level of PA performed by individuals during the past seven days in three categories, including walking, moderate activity, and vigorous activity [33]. The self-reported PA was converted into a metabolic equivalent (MET), where a higher MET indicates a higher level of PA. The IPAQ has shown good test-retest reliability, indicated by an intraclass correlation coefficient of 0.79 [34]. The Chinese version of IPAQ has also been used in weight stigma studies among Hong Kong populations [7, 35].

#### Eating behavior

The Three-Factor Eating Questionnaire-Revised 18-item version (TFEQ-R18) developed by Karlsson et al. [36] was used to assess three aspects of eating behavior: cognitive restraint (which indicates how much individuals intentionally limit their food intake to control body weight), uncontrolled eating (which indicates the difficulty in regulating consumption due to hunger or external food triggers), and emotional eating (which indicates the propensity to eat as a way to cope with negative emotions). Participants respond to each item using a Likert scale, and scores are converted to a 0–100 scale for each category, where higher scores signify a stronger tendency towards the particular eating behavior [37]. An example item is “*Sometimes when I start eating*,* I just can’t seem to stop.*” The Chinese version of TFEQ-R18 has also been used in weight stigma studies among Hong Kong populations [8, 38]. In the present study, the internal consistency of the total scale and subscales, assessed. by Cronbach’s alpha, ranged from 0.76 to 0.88 across T_1_ to T_4_.

#### Perceived behavioral control of physical activity (PBC-PA)

The PBC-PA was adopted from previous physical activity studies and is a subscale of the Theory of Planned Behavior Scale [7, 35, 39]. The subscale includes four 7-point items designed to assess perceived behavioral control related to PA. An example item is “*To what extent do you see yourself as being capable of exercising at least 30 minutes*,* 3 days in the next week?*” In the present study, the scale’s internal consistency as assessed by Cronbach’s alpha ranged from 0.83 to 0.84 across T_1_ to T_4_.

#### Perceived behavioral control of avoiding eating behaviors (PBC-AEB)

The perceived behavioral control of avoiding inappropriate eating behaviors was adopted from a previous weight stigma and eating behavior study, and is a subscale of the Theory of Planned Behavior Scale [8, 39]. The subscale included four 7-point items designed to assess perceived behavioral control related to avoiding inappropriate eating behaviors. An example item is “*How much personal control do you feel you have over whether you avoid inappropriate eating behaviors in the next week?*” In the present study, the scale’s internal consistency as by Cronbach’s alpha, ranged from 0.86 to 0.90 across T_1_ to T_4_.

#### Psychological distress

The 14-item Hospital Anxiety and Depression Scale (HADS), developed by Zigmond and Snaith [40], was used to assess psychological distress, particularly in relation to anxiety and depression. The instrument consists of two subscales, each containing seven items, and the total scores reflect the level of distress; higher scores signify increased psychological distress. An example item is “*Worrying thoughts go through my mind.*” The Chinese version of the HADS has shown acceptable psychometric properties [41]. In the present study, Cronbach’s α values for the HADS were between 0.82 and 0.87 across T_1_ to T_4_.

### Procedure

Participants completed the survey on an online survey platform hosted by the university and accessed through a QR code. Before completing the survey, participants were asked to read information about the study and their rights. They provided informed consent by selecting an “Agree” icon before accessing the survey. Follow-up surveys were distributed to participants through email, and text reminders were pushed to their mobile devices. Data were collected across four-month intervals. The initial baseline survey took place from March to June 2021. The second wave was conducted from July to October 2021, the third from November 2021 to February 2022, and the fourth from March to June 2022. For instance, a participant who enrolled in March 2021 would have subsequent data collection waves in July 2021, November 2021, and March 2022. A participant who enrolled in June 2021 would have their respective waves in October 2021, February 2022, and June 2022.

### Data analysis

Demographic characteristics of participants were summarized using descriptive statistics. Standardized scale scores were computed at baseline and during each subsequent follow-up period. Pearson correlation coefficients were calculated to examine relationships among scores from the PWSS, WBIS, PBC-PA, PBC-AEB, HADS, IPAQ, and TFEQ-R18 across all four timepoints. Moreover, a directed acyclic graph (DAG) showing the proposed associations between the studied variables can be found in the Supplementary Materials (Figure S1). There were no demographic differences between participants who dropped out at any point and those who remained, in terms of age (*t*[343] = -0.79, *p* = 0.430), gender (χ^2^ = 0.017 [1], *p* = 0.897), or BMI (*t*[343] = -1.207, *p* = 0.228).

To explore the longitudinal associations among variables, parallel-process latent growth curve model (PP-LGCM) was employed. This approach investigated the growth trajectories of the variables across time by estimating latent growth curves for each variable, estimating their initial levels (intercepts) and rates of change (slopes). Through this method, the covariation between the growth processes of different variables was analyzed, providing insight into their dynamic relationships over the study period.

#### PP-LGCM

Using the four repeated measures, the model estimated the latent intercept (initial level) and latent slope (growth). Consequently, latent intercepts and slopes were included for PWS, WRSS, PBC-PA, PBC-AEB, psychological distress, PA, cognitive restraints, emotional eating, uncontrolled eating, and BMI. The model specifically assessed (i) how the latent intercept of PWS influenced the latent intercept of WRSS, psychological distress, PA, cognitive restraints, emotional eating, uncontrolled eating, and BMI; (ii) how the latent intercept of WRSS affected the latent intercepts of PBC-PA, psychological distress, PA, cognitive restraints, emotional eating, uncontrolled eating, and BMI; (iii) how the latent intercept of PBC-PA and PBC-AEB impacted the latent intercepts of psychological distress and corresponding behaviors; (iv) how the latent intercept of psychological distress was associated with the latent intercept of four behaviors; and (v) how the latent intercept of four behaviors was associated with the latent intercept of BMI. Moreover, the model evaluated the relationships of the latent slopes of the variables as aforementioned, similar to the relationships between latent intercepts described above. Additionally, age and sex were included as time-invariant covariates, while the perceived impact of COVID-19 on PA and eating during each wave served as time-varying covariates for corresponding behaviors and PBC in the model.

#### RI-CLPM for the temporal association between WRSS and BMI

Given the uncertainty surrounding the potential vicious cycle between WRSS and BMI, a random intercept cross-lagged panel model (RI-CLPM) was used to assess it. This model examined whether earlier WRSS levels correlated with later BMI measurements, and if earlier BMI levels correlated with subsequent WRSS levels. It assessed the temporal relationships between WRSS and BMI over four timepoints, from T_1_ to T_4_ (Figure S2).

The PP-LGCM and RI-CLPM were deemed to be supported based on the incremental fit indices, specifically the comparative fit index (CFI) and the Tucker-Lewis index (TLI), both greater than 0.9, as well as the absolute fit indices, namely the standardized root mean square residual (SRMR) and the root mean square error of approximation (RMSEA) below 0.10 [42–44]. SPSS 29.0.2 (IBM Corp., Armonk, NY) was used to perform descriptive statistics, Pearson’s correlation of demographic data and scale scores’ RM-ANCOVA. R with the lavaan package [45] was used to perform the PP-LGCM and RI-CLPM. All of the models used maximum likelihood estimation with robust standard errors (MLR) and applied full information maximum likelihood (FIML) imputation to address missing data [46, 47].

## Results

### Descriptive statistics

The participants had a mean age of 22.94 years (SD = 3.33), and a mean BMI of 21.15 (SD = 3.22). Based on the cutoff for Asian adults [48], 21.2% of participants were classified as overweight or having obesity (BMI ≥ 23). Among all participants, nearly two-thirds were females (*n* = 218; 63.2%). At T_1_, the WBIS score showed 101 participants in the high WRSS group. Regarding COVID-19’s impact on PA levels, 62.6% reported decreases, 17.7% increases, and 19.7% no change. For eating behaviors, 28.4% ate less, 26.4% ate more, and 45.2% reported no change. BMI means were 21.13 (SD = 3.44) at T_2_, 21.32 (SD = 3.34) at T_3_, and 21.16 (SD = 3.25) at T_4_ (Table 1).

**Table 1:** Demographic information and descriptive statistics of the participants across the time

	Time 1 (*N* = 345)	Time 2 (*n* = 247-253)^a^	Time 3 (*n* = 228-233)^a^	Time 4 (*n* = 234)
Gender, *n* (%)				
Male	127 (36.8)	92 (36.4)	84 (36.1)	82 (35.0)
Female	218 (63.2)	161 (63.6)	149 (63.9)	152 (65.0)
Age (in years), *M* (*SD*)	22.94 (3.33)	23.07 (3.34)	23.45 (3.35)	23.87 (3.40)
Weight (kg), *M* (*SD*)	57.93 (11.61)	57.67 (11.61)	58.06 (11.68)	57.47 (11.35)
Body Mass Index (BMI), *M* (*SD*)	21.15 (3.22)	21.13 (3.44)	21.32 (3.34)	21.16 (3.25)
Weight group, *n* (%)				
Non-overweight (BMI < 23)	272 (78.8)	197 (77.9)	177 (76.0)	182 (77.8)
Overweight (BMI ≥ 23)	73 (21.2)	56 (22.1)	56 (24.0)	52 (22.2)
Perceived Weight Stigma Scale, *M* (*SD*)	1.24 (2.00)	1.29 (2.07)	1.13 (2.07)	1.06 (2.12)
Weight Bias Internalization Scale, *M* (*SD*)	2.46 (0.77)	2.44 (0.74)	2.42 (0.75)	2.42 (0.73)
Hospital Anxiety and Depression Scale, *M* (*SD*)	13.14 (6.03)	12.53 (5.94)	12.26 (6.52)	12.94 (6.74)
Perceived behavioral control toward PA, *M* (*SD*)	4.64 (1.31)	4.48 (1.26)	4.54 (1.21)	4.47 (1.21)
International Physical Activity Questionnaire, *M* (*SD*)	1955.49 (2442.70)	2495.21 (2600.13)	2250.01 (3137.30)	2205.66 (2882.06)
Perceived behavioral control toward avoiding EB, *M* (*SD*)	5.17 (1.21)	5.09 (1.28)	5.23 (1.13)	5.24 (1.23)
Three–Factor Eating Questionnaire–Revised 18–item version, *M* (*SD*)				
Cognitive restraint	43.67 (19.28)	44.02 (18.93)	44.37 (16.72)	43.40 (17.13)
Uncontrolled eating	37.21 (17.86)	35.76 (17.95)	34.83 (18.58)	33.54 (18.01)
Emotional eating	42.25 (25.07)	40.74 (24.11)	41.30 (25.46)	40.22 (24.61)
Perceived impact of COVID-19 on PA, *n* (%)				
More PA	61 (17.7)	44 (17.8)	22 (9.6)	37 (15.8)
Same	68 (19.7)	121 (49.0)	107 (46.9)	52 (22.2)
Less PA	216 (62.6)	82 (33.2)	99 (43.4)	145 (62.0)
Perceived impact of COVID-19 on eating, *n* (%)				
More eating	91 (26.4)	36 (14.6)	44 (19.3)	67 (28.6)
Same	156 (45.2)	170 (68.8)	154 (67.5)	111 (47.4)
Less eating	98 (28.4)	41 (16.6)	30 (13.2)	56 (23.9)

*PA* physical activity, *EB* eating behaviors. ^a^Some participants had missing data, such as not reporting the perceived impact of COVID-19.

Tables 2 and 3, and 4 illustrate the relationships among variables. Overall, both PWS and WRSS had a significant positive correlation with psychological distress, while WRSS had a negative correlation with PBC-PA and PBC-AEB. PBC-PA was positively associated with psychological distress and PA levels. On the other hand, PBC-AEB was negatively correlated with both uncontrolled and emotional eating but positively correlated with cognitive restraint. Physical activity was positively correlated with cognitive restraint and negatively correlated with uncontrolled eating and emotional eating.

**Table 2:** Correlation between perceived weight stigma, weight-related self-stigma, psychological distress, perceived behavioral control for physical activity, and physical activity

	*r*											
Variables	PWS1	PWS2	PWS3	PWS4	WRSS1	WRSS2	WRSS3	WRSS4	PD1	PD2	PD3	PD4
PWS1	–											
PWS2	0.53***	–										
PWS3	0.50***	0.73***	–									
PWS4	0.59***	0.70***	0.69***	–								
WRSS1	0.48***	0.36***	0.35***	0.39***	–							
WRSS2	0.44***	0.43***	0.43***	0.44***	0.80***	–						
WRSS3	0.44***	0.41***	0.46***	0.49***	0.78***	0.82***	–					
WRSS4	0.43***	0.44***	0.43***	0.50***	0.76***	0.79***	0.82***	–				
PD1	0.33***	0.34***	0.34***	0.36***	0.28***	0.25***	0.37***	0.37***	–			
PD2	0.29***	0.39***	0.34***	0.36***	0.24***	0.27***	0.36***	0.35***	0.68***	–		
PD3	0.26***	0.37***	0.39***	0.39***	0.31***	0.32***	0.47***	0.41***	0.70***	0.76***	–	
PD4	0.31***	0.34***	0.34***	0.40***	0.23***	0.19**	0.31***	0.36***	0.69***	0.72***	0.77***	–
PBC-PA1	-0.01	-0.09	-0.04	-0.02	-0.17**	-0.15*	-0.10	-0.12	-0.20***	-0.21***	-0.17**	-0.07
PBC-PA2	-0.05	-0.12	-0.11	-0.13	-0.18**	-0.18**	-0.17*	-0.14*	-0.25***	-0.22***	-0.21**	-0.13
PBC-PA3	-0.03	-0.06	-0.09	-0.14*	-0.24***	-0.25***	-0.23***	-0.20**	-0.20**	-0.17**	-0.23***	-0.15*
PBC-PA4	0.001	-0.19**	-0.19**	-0.16*	-0.17*	-0.21**	-0.20**	-0.18**	-0.24***	-0.26***	-0.23***	-0.25***
PA1	0.17**	0.06	0.03	0.07	0.05	0.003	0.06	0.03	-0.05	-0.04	0.02	0.05
PA2	0.07	0.09	-0.02	-0.08	0.03	-0.01	0.03	-0.05	-0.09	-0.09	-0.09	-0.11
PA3	0.02	0.04	-0.09	-0.09	0.05	-0.01	0.03	-0.02	0.004	-0.05	-0.08	-0.03
PA4	-0.03	0.12	0.06	-0.04	0.02	-0.02	0.02	0.01	-0.01	-0.05	0.03	-0.06

	*r*							
Variables	PBC-PA1	PBC-PA2	PBC-PA3	PBC-PA4	PA1	PA2	PA3	PA4
PBC-PA1	–							
PBC-PA2	0.63***	–						
PBC-PA3	0.63***	0.61***	–					
PBC-PA4	0.50***	0.62***	0.66***	–				
PA1	0.33***	0.34***	0.29***	0.20**	–			
PA2	0.13*	0.13*	0.16*	0.09	0.39***	–		
PA3	0.25***	0.23***	0.31***	0.26***	0.30***	0.36***	–	
PA4	0.08	0.11	0.08	0.16*	0.26***	0.52***	0.28***	–

*PWS* perceived weight stigma, *WRSS* weight-related self-stigma, *PD* psychological distress, *PA* physical activity, *PBC-PA* perceived behavioral control for physical activity. The numbers (1 to 4) that follow the variable name indicate the variable at Timepoint 1 to Timepoint 4. *p < 0.05. **p < 0.01. ***p < 0.001.

**Table 3:** Correlation between perceived weight stigma, weight-related self-stigma, psychological distress, perceived behavioral control for avoiding eating behaviors, and the three eating behaviors

	*r*											
Variables	PWS1	PWS2	PWS3	PWS4	WRSS1	WRSS2	WRSS3	WRSS4	PD1	PD2	PD3	PD4
PBC-AEB1	-0.16**	-0.06	-0.11	-0.16*	-0.25***	-0.17**	-0.29***	-0.29***	-0.27***	-0.21***	-0.25***	-0.32***
PBC-AEB2	-0.07	-0.13*	-0.04	-0.12	-0.26***	-0.23***	-0.26***	-0.26***	-0.23***	-0.25***	-0.21**	-0.23***
PBC-AEB3	-0.07	-0.12	-0.12	-0.18**	-0.26***	-0.22**	-0.28***	-0.25***	-0.17**	-0.18**	-0.24***	-0.27***
PBC-AEB4	-0.13*	-0.15*	-0.15*	-0.25***	-0.26***	-0.19**	-0.27***	-0.31***	-0.29***	-0.27***	-0.24***	-0.34***
CR1	0.17**	0.06	0.02	0.007	0.27***	0.19**	0.20**	0.16*	0.06	-0.02	0.02	0.03
CR2	0.07	0.14*	0.09	0.06	0.22***	0.28***	0.23***	0.18**	-0.07	-0.003	0.03	-0.008
CR3	0.05	0.06	0.02	-0.04	0.20**	0.17*	0.21**	0.14*	-0.03	-0.02	-0.009	-0.05
CR4	0.02	0.02	0.02	0.02	0.23***	0.26***	0.22**	0.25***	0.02	0.02	0.08	0.06
UE1	0.21***	0.16*	0.20**	0.17*	0.30***	0.24***	0.29***	0.28***	0.25***	0.26***	0.25***	0.32***
UE2	0.26***	0.23***	0.23***	0.28***	0.30***	0.31***	0.38***	0.34***	0.24***	0.30***	0.25***	0.27***
UE3	0.28***	0.20**	0.21**	0.26***	0.39***	0.34***	0.43***	0.39***	0.24***	0.25***	0.30***	0.31***
UE4	0.25***	0.14*	0.18**	0.19**	0.38***	0.34***	0.40***	0.42***	0.25***	0.26***	0.25***	0.34***
EE1	0.21***	0.19**	0.26***	0.16*	0.31***	0.28***	0.29***	0.28***	0.19***	0.15*	0.19**	0.22***
EE2	0.21***	0.20**	0.20**	0.20**	0.25***	0.32***	0.28***	0.27***	0.15*	0.20**	0.19**	0.14*
EE3	0.17*	0.18**	0.20**	0.17*	0.41***	0.34***	0.39***	0.36***	0.15*	0.11	0.21**	0.16*
EE4	0.17*	0.17*	0.23***	0.16*	0.26***	0.27***	0.32***	0.33***	0.21**	0.15*	0.20**	0.28***

	*r*							
Variables	PBC-AEB1	PBC-AEB2	PBC-AEB3	PBC-AEB4	CR1	CR2	CR3	CR4
PBC-AEB1	–							
PBC-AEB2	0.54***	–						
PBC-AEB3	0.53***	0.57***	–					
PBC-AEB4	0.58***	0.62***	0.62***	–				
CR1	0.12*	0.10	0.03	0.12	–			
CR2	0.19**	0.16*	0.03	0.20**	0.76***	–		
CR3	0.17**	0.19**	0.08	0.14*	0.72***	0.73***	–	
CR4	0.07	0.11	0.009	0.10	0.64***	0.70***	0.69***	–
UE1	-0.38***	-0.27***	-0.34***	-0.42***	0.006	-0.06	0.002	0.05
UE2	-0.30***	-0.28***	-0.29***	-0.37***	0.03	-0.006	-0.02	-0.04
UE3	-0.40***	-0.31***	-0.45***	-0.40***	0.001	-0.01	-0.002	-0.01
UE4	-0.37***	-0.32***	-0.36***	-0.44***	0.05	-0.002	0.03	0.09
EE1	-0.32***	-0.21**	-0.30***	-0.36***	0.03	-0.06	-0.07	0.02
EE2	-0.26***	-0.21***	-0.25***	-0.27***	0.08	0.04	0.01	-0.05
EE3	-0.33***	-0.20**	-0.34***	-0.28***	0.07	0.03	0.05	0.04
EE4	-0.30***	-0.21**	-0.22**	-0.39***	0.03	-0.01	-0.02	0.11

	*r*							
Variables	UE1	UE2	UE3	UE4	EE1	EE2	EE3	EE4
CR1								
CR2								
CR3								
CR4								
UE1	–							
UE2	0.71***	–						
UE3	0.71***	0.76***	–					
UE4	0.71***	0.73***	0.77***	–				
EE1	0.60***	0.50***	0.51***	0.58***	–			
EE2	0.48***	0.67***	0.55***	0.51***	0.68***	–		
EE3	0.46***	0.54***	0.68***	0.54***	0.69***	0.70***	–	
EE4	0.40***	0.44***	0.47***	0.66***	0.68***	0.60***	0.68***	–

*PWS* perceived weight stigma, *WRSS* weight-related self-stigma, *PD* psychological distress, *PBC-AEB* perceived behavioral control for avoiding eating behaviors, *CR* cognitive restraint, *UE* uncontrolled eating, *EE* emotional eating. The numbers (1 to 4) that follow the variable name indicate the variable at Time 1 to 4. *p < 0.05. **p < 0.01. ***p < 0.001.

**Table 4:** Correlation between physical activity and the three eating behaviors

	*r*			
Variables	PA1	PA2	PA3	PA4
CR1	0.13*	0.13*	0.22***	0.10
CR2	0.20**	0.10	0.20**	-0.003
CR3	0.12	0.05	0.27***	0.01
CR4	0.14*	0.01	0.23***	0.03
UE1	0.08	-0.03	0.05	-0.12
UE2	0.06	-0.01	-0.03	-0.15*
UE3	0.12	0.05	-0.06	-0.10
UE4	0.13	-0.04	-0.002	-0.13*
EE1	0.06	-0.03	0.05	-0.13*
EE2	0.03	-0.04	-0.06	-0.11
EE3	0.08	0.02	-0.06	-0.09
EE4	0.09	-0.08	-0.03	-0.17**

*PA* physical activity, *CR* cognitive restraint, *UE* uncontrolled eating, *EE* emotional eating. The numbers (1 to 4) that follow the variable name indicate the variable at Timepoint 1 to Timepoint 4. *p < 0.05. **p < 0.01. ***p < 0.001.

### Results of the latent growth model analysis

**Fig. 2:**
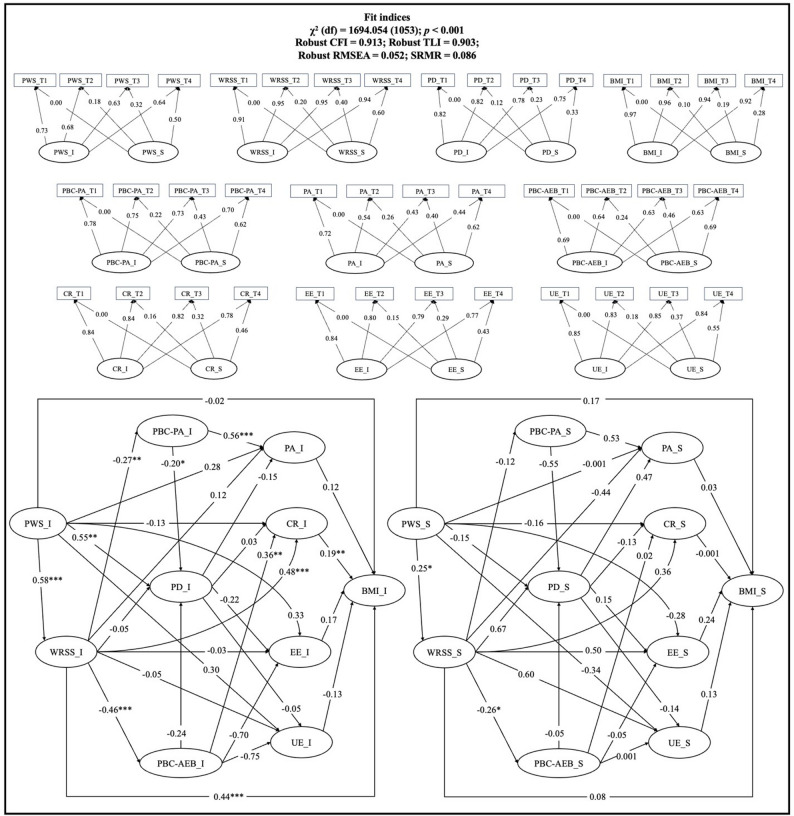
The parallel-process latent growth curve model. *BMI* body mass index, *PWS* perceived weight stigma, *WRSS* weight-related self-stigma, *PD* psychological distress, *PA* physical activity, *PBC-PA* perceived behavioral control for physical activity, *PBC-AEB* perceived behavioral control for avoiding eating behaviors, *UE* uncontrolled eating, *CR* cognitive restraint, *EE* emotional eating, *T1* Timepoint 1, *T2* Timepoint 2, *T3* Timepoint, *T4* Timepoint 4. The upper part of the figure illustrates each pair of the latent intercept (I) and latent slope (S) of the variables, the lower left part illustrates the parallel process between latent intercepts, and the lower right part illustrates the parallel process between latent slopes. The latent intercept represents the initial level of a variable, and the latent slope represents the growth (i.e., rate of change) of a variable. All path coefficients presented are standardized coefficients. *p < 0.05. **p < 0.01. ***p < 0.001

PP-LGCM showed a satisfactory fit: χ^2^ (df) = 1694.054 (1053); *p* < 0.001; robust CFI = 0.913; robust TLI = 0.903; robust RMSEA = 0.052; SRMR = 0.086 (Fig. 2). For path coefficients, the initial level (intercept) of PWS had a significant relationship with the intercept of WRSS (standardized coefficient [β] = 0.58; *p* < 0.001) and psychological distress (β = 0.55; *p* = 0.001); the intercept of WRSS had a significant relationship with the intercept of PBC-PA (β = -0.27; *p* = 0.004), PBC-AEB (β = -0.46; *p* < 0.001), cognitive restraint (β = 0.48; *p* < 0.001), and BMI (β = 0.44; *p* < 0.001) but had no significant relationship with psychological distress (*p* = 0.791), emotional eating (*p* = 0.922), and uncontrolled eating (*p* = 0.869); the intercept of PBC-PA had a significant relationship with the intercept of psychological distress (β = -0.20; *p* = 0.017) and PA (β = 0.56; *p* < 0.001); the intercept of PA had no significant relationship with the intercept of BMI (*p* = 0.122); the intercept of PBC-AEB had a significant relationship with the intercept of cognitive restraint (β = 0.36; *p* = 0.003); the intercept of cognitive restraint had a significant relationship with the intercept of BMI (β = 0.19; *p* = 0.006). Moreover, the growth trajectory (slope) of PWS had a significant relationship with the slope of the WRSS (β = 0.25; *p* = 0.015); the slope of WRSS had a significant relationship with the slope of PBC-AEB (β = -0.26; *p* = 0.042). There were connections found only between some pairs of slopes: the relationship between PWS and WRSS, and WRSS and PBC-AEB. No changes in any other variables were related to changes in BMI.

### Results of the RI-CLPM on the relationship between WRSS and BMI

**Fig. 3:**
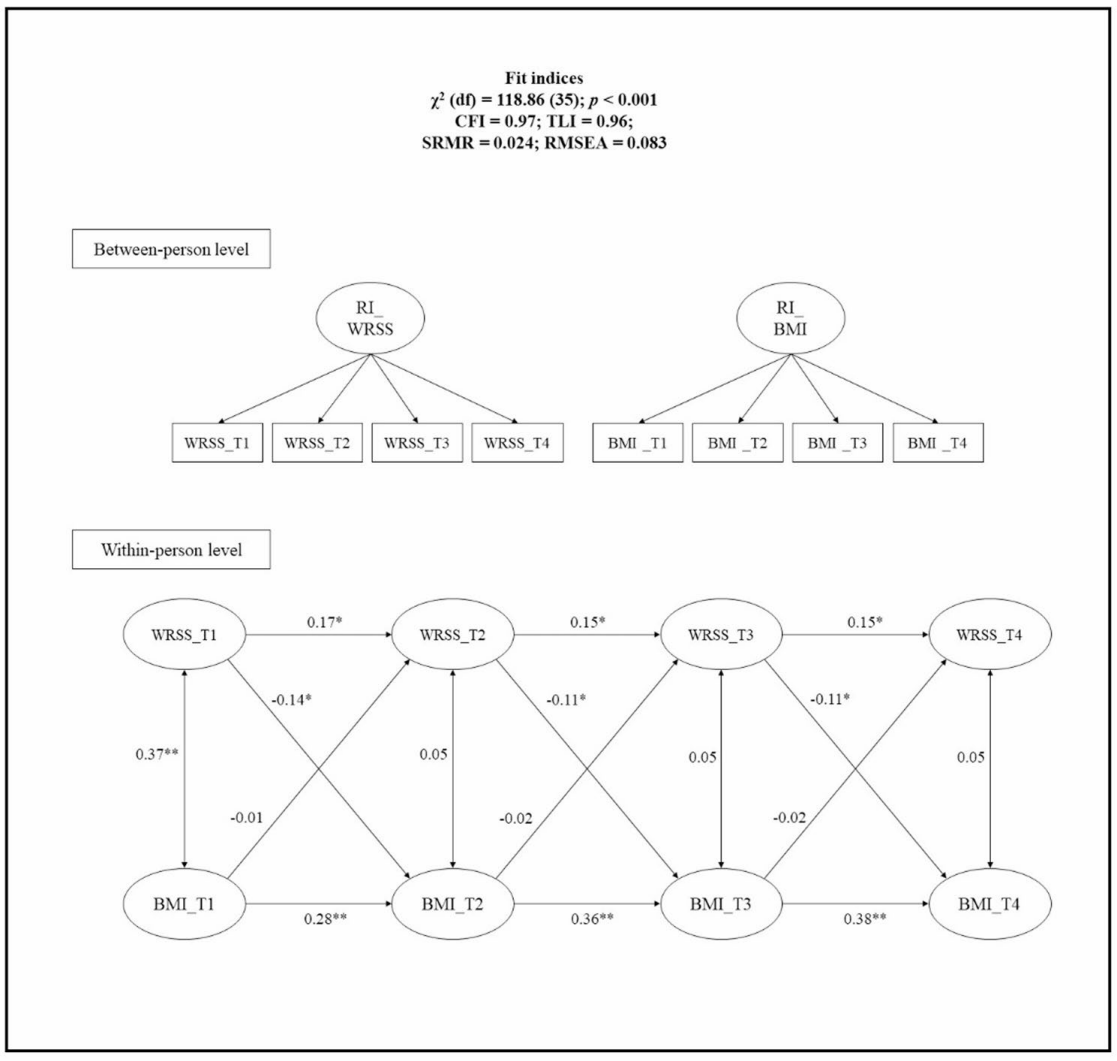
The random intercept cross-lagged panel model on the relationship between WRSS and BMI. *WRSS* weight-related self-stigma, *BMI* body mass index, *T1* Timepoint 1, *T2* Timepoint 2, *T3* Timepoint, *T4* Timepoint 4. *p < 0.05. **p < 0.01

The proposed RI-CLPM (Fig. 3) showed satisfactory fits (CFI = 0.97, TLI = 0.96, RMSEA = 0.083, and SRMR = 0.024). There were significant associations between prior WRSS and following BMI across various timepoints: from T_1_ to T_2_ (β = -0.14; *p* = 0.032), from T_2_ to T_3_ (β = -0.11; *p* = 0.032), and from T_3_ to T_4_ (β = -0.11; *p* = 0.032). In contrast, there was no significant associations between prior BMI and following WRSS during T_1_ to T_2_ (β = -0.01; *p* = 0.806), T_2_ to T_3_ (β = -0.02; *p* = 0.806), and T_3_ to T_4_ (β = -0.02; *p* = 0.806). This indicated that while increased WRSS was linked to a lower subsequent BMI, BMI did not influence the following WRSS.

## Discussion

The present longitudinal study explored how perceived stigma, self-stigma, perceived behavioral control, psychological distress, PA, and BMI were associated over a one-year time period. Using PP-LGCM, significant relationships over time were found only between PWS, WRSS, and PBC-AEB. Moreover, RI-CLPM was constructed to understand bi-directional relationships, showing a negative relationship between WRSS and future BMI, whereas future WRSS showed no significant association with BMI. Therefore, the vicious cycle was not identified. In sum, H_1_ was supported, H_2_ was partially supported, and H_3_, H_4_, H_5_, H_6_, and H_7_ were not supported.

### Relationship between perceived stigma and self-stigma

The findings of the present study support existing literature on the relationship between perceived stigma and self-stigma over time. For example, a three-month longitudinal study conducted in Taiwan showed that changes in perceived weight stigma corresponded with changes in internalized weight stigma among university students [49]. The significant relationship observed in the present one-year study of a Hong Kong young adult sample suggested that as individuals become aware of their stigmatization, they are highly likely to internalize these distorted values, thereby resulting in greater self-stigma [1, 20]. In other words, the results align with the idea that perceived stigma shapes how individuals view themselves, reinforcing discriminatory attitudes [17]. This highlights the need to tackle societal stereotypes and biases to help prevent individuals from internalizing such negative perceptions about their weight.

### Relationships between weight stigma, perceived behavioral control, and psychological distress

A notable temporal relationship was found between weight self-stigma and perceived behavioral control. The present study’s findings indicated that individuals’ greater weight self-stigma was associated with a diminished sense of control over their behavior. This relationship is apparent not only in cross-sectional studies but also longitudinally, showing that enduring weight self-stigma contributes to consistently low perceived behavioral control. This suggests that weight self-stigma impacts an individual’s confidence in making positive changes to their health behaviors. The results align with findings from a cross-sectional study in the US, which demonstrated a relationship between weight self-stigma and self-efficacy in managing eating habits [50].

Moreover, the findings of the present study provide further evidence supporting that weight self-stigma is consistently associated with a reduction in perceived behavioral control. The literature indicates that self-stigmatization related to weight can reduce self-control, impacting various aspects such as attentional, behavioral, and emotional impulses [51]. More specifically, the perception of devaluation stemming from self-stigma is uncontrollable, which may exacerbate these effects and result in a decline in perceived behavioral control [51]. Moreover, research conducted across a 20-year time period in the US, demonstrated significant declines in both overall and general perceived control among young adults in comparison to their middle-aged counterparts [52]. Moreover, perceived daily control, defined as the ability to manage specific daily activities and stressors, experienced more pronounced declines than overall perceived control [52]. Consequently, the present study's findings, combined with evidence from the extant literature, indicate a need to tackle the issue of reduced perceived behavioral control due to self-stigma among young adults. Future research could explore, for instance, if programs aimed at reducing weight stigma can enhance the perceived control over weight management behaviors.

Moreover, a significant relationship was found between PWS and psychological distress, but only at the initial level. Research indicates that individuals facing weight stigma often experience mental health challenges, such as heightened depression and anxiety [53]. A meta-analysis by Alimoradi et al. [9] noted a scarcity of longitudinal studies exploring the link between weight stigma and psychological distress. The present study strengthens the evidence of this relationship, suggesting that weight stigma fosters negative self-perception and feelings of worthlessness, which can trigger or exacerbate mental health challenges. However, further research is needed to understand why the present study found no significant relationship between perceived behavioral control and psychological distress over time. Nevertheless, the results indicated that perceived weight discrimination contributed to the internalization of stigma and psychological distress. It is imperative to educate and eradicate weight stigma to prevent further societal harm. Future research should also explore methods and protective factors that prevent individuals from internalizing stigma and that help them to manage their psychological well-being effectively.

### Relationship between weight stigma and BMI

Recent literature has explored the longitudinal association between weight stigma and changes in body weight. Notable examples include two observational studies [49, 54] and one intervention study [55]. For instance, a study by Lin et al. [49] indicated that increased WRSS corresponded with an increase in BMI, implying that weight stigma impacts not only psychological well-being but also physiological outcomes. Nevertheless, a limitation was the three-month duration of the study [49]. The present study, conducted over a one-year period, yielded contrasting results. The findings from RI-CLPM indicated a negative association between WRSS and BMI in the subsequent measurements. In other words, higher levels of WRSS were associated with lower BMI, while lower levels of WRSS were associated with higher BMI.

These findings challenge the prevailing assumption that increased weight stigma contributes to greater body weight. However, the results may not fully address the complexity of this relationship because unexamined factors could potentially influence or clarify the observed negative association between weight-related stigma and weight changes. Future research should investigate the underlying mechanisms that may account for these outcomes. Lee et al. [54] indicated that weight stigma significantly correlated with heightened perceived stress and comfort eating behaviors. While comfort eating did not predict weight changes over subsequent four-month intervals, greater weight was associated with increased daily experiences of weight stigma during these periods [54].

In alignment with findings by Lee et al. [54], the present study’s PP-LGCM showed no significant longitudinal associations between eating behaviors (such as emotional and uncontrolled eating) and BMI. This may be attributed to the one-year duration with four timepoints being insufficient to detect weight changes. Moreover, Lee et al. [54] also found that weight was positively associated with subsequent weight stigma, even though the effect was minimal. Conversely, no relationship was found in the present study, which might be due to a different measurement of weight stigma. More specifically, Lee et al. [54] examined daily encounters with weight stigma, while the present study focused on the internalization of stigma.

Moreover, the long-process of internalization might be another factor to consider. It is possible that WRSS necessitates an extended duration for the internalization process. Consequently, identifying the changes within the present study’s time frame remain challenging. This underscores the need for longitudinal research over a longer duration to thoroughly investigate the relationship. Alternatively, it is also plausible that the level of WRSS may not continue to escalate, thereby indicating a static relationship. Indeed, the present study showed that the correlations of WRSS across different timepoints were approximately 0.8. Moreover, one study found that WRSS is stable over a one-month period among the Spanish general population, with a correlation of 0.88 [56]. Another study found temporal stability of WRSS from the third trimester to one month postpartum among pregnant women with higher weight [57].

On the other hand, research has examined the effects of WRSS on weight changes in a 72-week behavioral weight loss intervention [55]. The findings suggested that individuals with a greater reduction in WRSS also had greater weight loss in subsequent measurement. Sheynblyum et al. [55] did not examine the relationship between weight change and subsequent weight stigma. However, studies examining programs aimed at reducing weight or stigma seem to be an effective method for exploring this mutual relationship. Given that changes in weight and stigma tend to occur gradually, these weight reduction programs could help track these dynamics over time.

### Impact of COVID-19 on the study variables

One of the possible factors contributing to the non-significant results of the longitudinal relationships may have been the local circumstances of the COVID-19 pandemic in Hong Kong during the data collection period, which led to changes in individuals’ dietary habits and physical activities [25]. Notably, during that period, Hong Kong experienced the most severe effects of the pandemic [58]. Hong Kong experienced rapid changes in the pandemic’s seriousness, including social distancing, quarantine, restricted dining hours, and the closure of sports and fitness facilities [59–61]. The government took a flexible approach, adjusting COVID-19 measures based on the situation, leading to frequent changes over a few months [61, 62]. The infection, quarantine, and closure of sports venues, may have impacted PA and perceived behavioral control during that year, potentially altering habits. Although the survey explored COVID-19’s perceived impact on PA, adjusting for these changes is challenging. Additionally, dine-in service hours were restricted [61]. Moreover, worries about infection, and individuals’ habits of eating outside of their home may have decreased. During the COVID-19 pandemic, eating at home or alone could also have influenced eating patterns [63, 64] and potentially lead to maladaptive eating behaviors [65].

### Limitations and future directions

The present study has several limitations. First, all measures were based on self-report, which can introduce biases, such as recall bias in estimating the duration of PA and the effects of social desirability in responses regarding weight stigma and weight. Moreover, the sample (which was relatively small) may not have been representative of the broader population of young adults. The participants were mostly university students, whose lifestyles tend to be more similar to each other than those of other groups, such as working individuals. Additionally, the study did not inquire about participants’ employment status, leaving the student-to-worker ratio ambiguous. Moreover, as aforementioned, the one-year duration of the present longitudinal study may not have adequately addressed the impact of stigma internalization and weight changes. Future research should investigate the long-term effects over more extended periods. Because the study was conducted during the COVID-19 pandemic, physical activity, eating behaviors, and psychological distress might have been affected. Future studies in a more stable environment could help clarify these relationships. In addition, future research may consider adding the environmental impacts on weight stigma to identify how PWS and WRSS developed. A newly developed measure (the Weight Stigma Exposure Inventory) helps assess observed weight stigma from different sources such as the media [66–69], which might be useful in future studies.

## Conclusion

Using a one-year longitudinal survey, the present study identified temporal relationships among PWS, WRSS, perceived behavioral control, and psychological distress. However, the study did not provide additional evidence for a potential feedback loop involving weight stigma and weight change. The findings indicate that further research is necessary to determine if weight stigma may be influenced by weight gain. The findings support a clearer understanding of weight management and underscore the harmful impacts of weight stigma. The findings also showed significant issues regarding weight stigma in Hong Kong, highlighting the need for improvements in areas such as public education and stigma-reduction initiatives.

## Supplementary Information

Below is the link to the electronic supplementary material.

Supplementary Material 1.

## Data Availability

The datasets generated during and/or analyzed during the present study are available from the corresponding author upon reasonable request.

## Abbreviations

BMI: Body mass index
CFI: Comparative fit index
FIML: Full information maximum likelihood
HADS: Hospital Anxiety and Depression Scale
IPAQ: International Physical Activity Questionnaire
MLR: Maximum likelihood estimation with robust standard errors
PA: Physical activity
PBC-AEB: Perceived behavioral control for avoiding eating behaviors
PBC-PA: Perceived behavioral control for physical activity
PP-LGCM: Parallel-process latent growth curve model
PWS: Perceived weight stigma
PWSS: Perceived Weight Stigma Scale
RI-CLPM: Random intercept cross-lagged panel model
RM-ANCOVA: Repeated measures analysis of covariance
RMSEA: Root mean square error of approximation
SRMR: Standardized root mean square residual
TFEQ-R18: Three-Factor Eating Questionnaire-R18
TLI: Tucker-Lewis index
WBIS: Weight Bias Internalization Scale
WRSS: Weight-related self-stigma
