# Supplementary material for: Exploring how weight stigma relates to psychological distress, physical activity, and eating behaviors over time: a longitudinal study among young adults in Hong Kong
Source: J Eat Disord. 2026 Jan 19;14:50. doi: 10.1186/s40337-026-01525-w (PMC12896354; doi:10.1186/s40337-026-01525-w)
Supplement: Supplementary file 1 — Supplementary Material 1. [file 40337_2026_1525_MOESM1_ESM.docx]

Supplementary Material

Figure S1. Directed Acyclic Graph (DAG) of the associations between the studied variables.


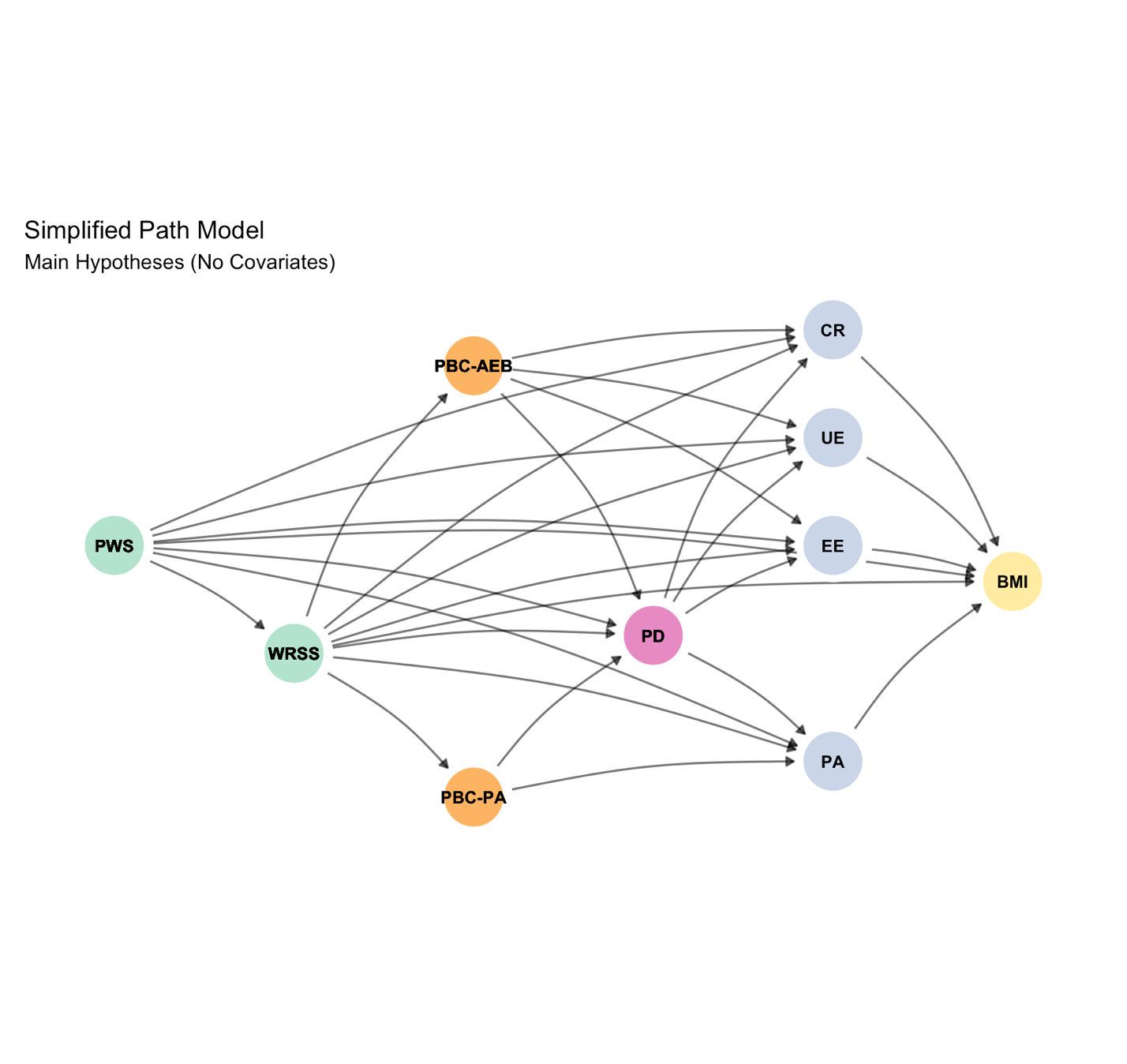


*Note.* BMI = body mass index; PWS = perceived weight stigma; WRSS = weight-related self-stigma; PD = psychological distress; PA = physical activity; PBC-PA = perceived behavioral control for physical activity; PBC-AEB = perceived behavioral control for avoiding eating behaviors; UE = uncontrolled eating; CR = cognitive restraint; EE = emotional eating.

Figure S2. *The Parallel-Process Latent Growth Curve Model with the paths from BMI to PWS and WRSS*


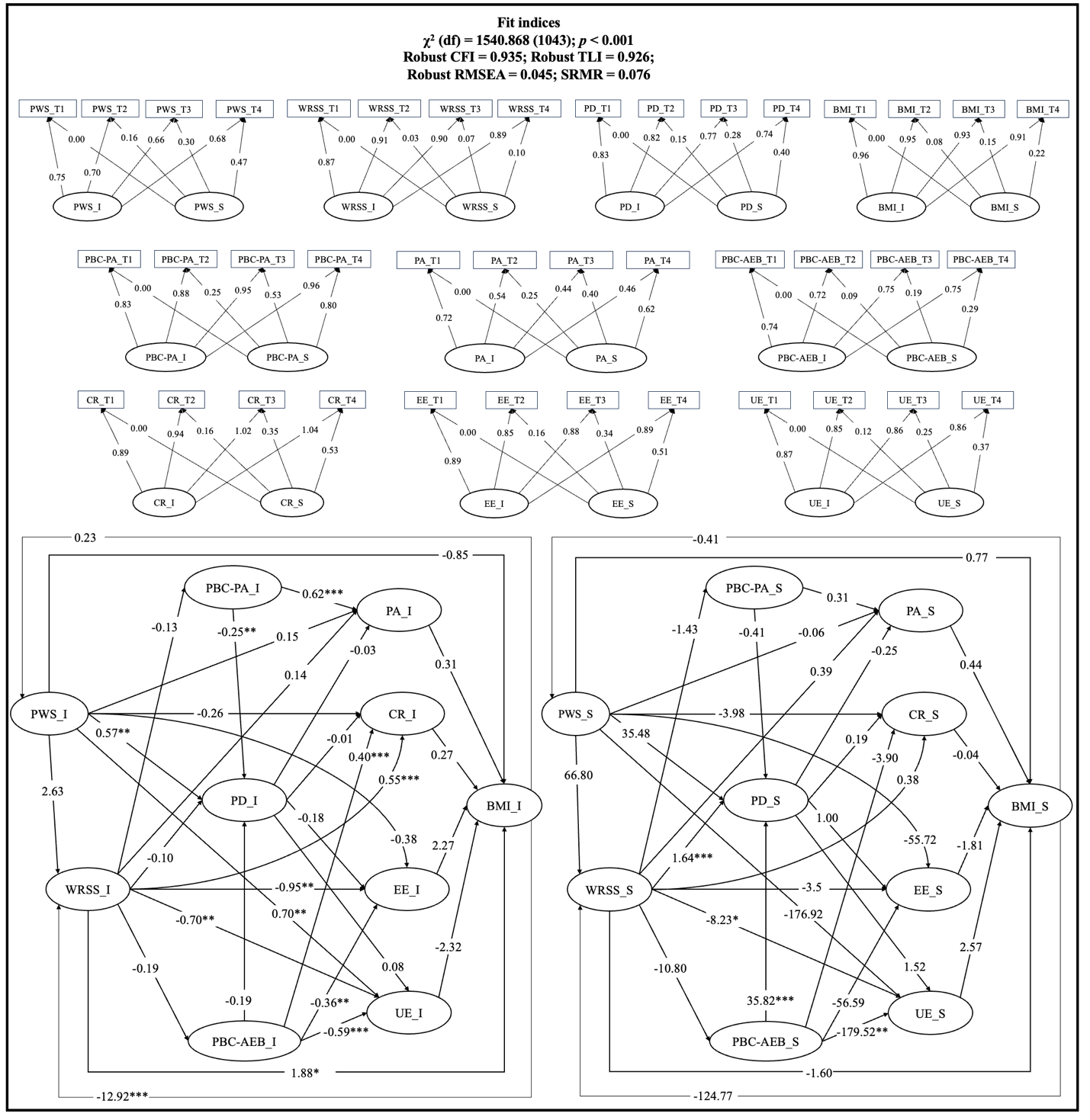


*Note*. BMI = body mass index; PWS = perceived weight stigma; WRSS = weight-related self-stigma; PD = psychological distress; PA = physical activity; PBC-PA = perceived behavioral control for physical activity; PBC-AEB = perceived behavioral control for avoiding eating behaviors; UE = uncontrolled eating; CR = cognitive restraint; EE = emotional eating; T1 = Time 1; T2 = Time 2; T3 = Time; T4 = Time 4. The upper part of the figure illustrates each pair of the latent intercept (I) and latent slope (S) of the variables, the lower left part illustrates the parallel process between latent intercepts, and the lower right part illustrates the parallel process between latent slopes. The latent intercept represents the initial level of a variable, and the latent slope represents the growth (i.e., rate of change) of a variable. All path coefficients presented are standardized coefficients. **p* < 0.05. ***p* < 0.01. ****p* < 0.001.
